# A Novel Assay to Trace Proliferation History In Vivo Reveals that Enhanced Divisional Kinetics Accompany Loss of Hematopoietic Stem Cell Self-Renewal

**DOI:** 10.1371/journal.pone.0003710

**Published:** 2008-11-12

**Authors:** Jens M. Nygren, David Bryder

**Affiliations:** Stem Cell Aging, Department of Experimental Medical Science, Lund University, Lund, Sweden; University of Minnesota, United States of America

## Abstract

**Background:**

The maintenance of lifelong blood cell production ultimately rests on rare hematopoietic stem cells (HSCs) that reside in the bone marrow microenvironment. HSCs are traditionally viewed as mitotically quiescent relative to their committed progeny. However, traditional techniques for assessing proliferation activity *in vivo*, such as measurement of BrdU uptake, are incompatible with preservation of cellular viability. Previous studies of HSC proliferation kinetics *in vivo* have therefore precluded direct functional evaluation of multi-potency and self-renewal, the hallmark properties of HSCs.

**Methodology/Principal Findings:**

We developed a non-invasive labeling technique that allowed us to identify and isolate candidate HSCs and early hematopoietic progenitor cells based on their differential *in vivo* proliferation kinetics. Such cells were functionally evaluated for their abilities to multi-lineage reconstitute myeloablated hosts.

**Conclusions:**

Although at least a few HSC divisions *per se* did not influence HSC function, enhanced kinetics of divisional activity in steady state preceded the phenotypic changes that accompanied loss of HSC self-renewal. Therefore, mitotic quiescence of HSCs, relative to their committed progeny, is key to maintain the unique functional and molecular properties of HSCs.

## Introduction

The generation of blood cells is a hierarchical developmental process that emanates from rare hematopoietic stem cells (HSCs) that reside in the bone marrow [1]. Previous studies have demonstrated that HSCs proliferate *in vivo* with slow kinetics [2], [3], [4]. Upon differentiation, HSCs irreversibly enter progenitor cell stages characterized by extensive proliferation at the expense of their self-renewal potential [5]. Although this generic model has been relatively undisputed, mechanisms must exist that allow for modulation of the proliferative properties of HSC. For example, HSC numbers expand dramatically following transplantation and it is well established that cytostatic regimens and cytokines can induce their rapid cycling [6], [7]. Furthermore, HSCs proliferate to a higher extent in fetal development [8] through what appears to be an intrinsic control mechanism [9]. Defining the mechanisms that govern these events is of great interest; such information could be used for therapeutic benefit as well as to increase the current understanding of HSC self-renewal in both normal and aberrant hematopoiesis.

In an attempt to overcome obstacles associated with traditional techniques aimed at investigating HSC proliferation dynamics, we have developed a technique that permits the evaluation of HSC proliferation *in vivo* over extended time periods. Intravenous injection of an N-hydroxysulfosuccinimide biotin derivative (referred to hereafter as biotin) effectively labeled the membrane proteins of all hematopoietic cells in peripheral blood, spleen, thymus, and bone marrow. Upon proliferation, labeled membrane proteins were distributed roughly equally among daughter cells, causing a linear reduction in biotin label. The technique therefore allowed for assessment of the *in vivo* proliferative history of individual candidate HSCs and progenitor cells over the course of several weeks. A unique feature of this approach was that streptavidin-based detection of the biotin probe allowed for preservation of cell viability throughout analysis. Therefore, prospectively isolated HSCs with different proliferation history could be subjected to functional and molecular investigation. With this novel approach, we could confirm previous studies demonstrating that functionally defined HSCs are slow dividing compared to their down-stream progenitor cell subsets [3], [5], although long-term HSC dormancy was, if present at all, a very rare phenomenon.

In our studies, a subset of cells within a candidate HSC compartment was found to divide with slower kinetics and harbor improved *in vivo* multi-lineage competitive repopulation and self-renewal capacities in steady state. These slowly dividing HSCs represented the most primitive subset of HSCs, and enhancement of their proliferation kinetics in steady state, but not after cytostatic stress, marked a primary event associated with loss of HSC self-renewal.

## Results

### Tracing proliferation history of candidate hematopoietic stem cells using an *in vivo* biotinylation strategy

Intravenous administration of esterized versions of biotin is a well-established technique to label erythrocytes and platelets *in vivo* [10], [11]. The life span of such non-dividing cells can then be determined by enumerating the fraction of label-retaining cells in circulation at various time points after biotinylation. Importantly, such labeling is characterized by remarkable stability, highlighted by the ability to trace biotin-labeled erythrocytes more than 100 days after labeling [12]. We speculated that biotin labeling could also be used to label other cell types. Of particular interest to us was its labeling efficiency in mitotically competent cells.

**Figure 1:**
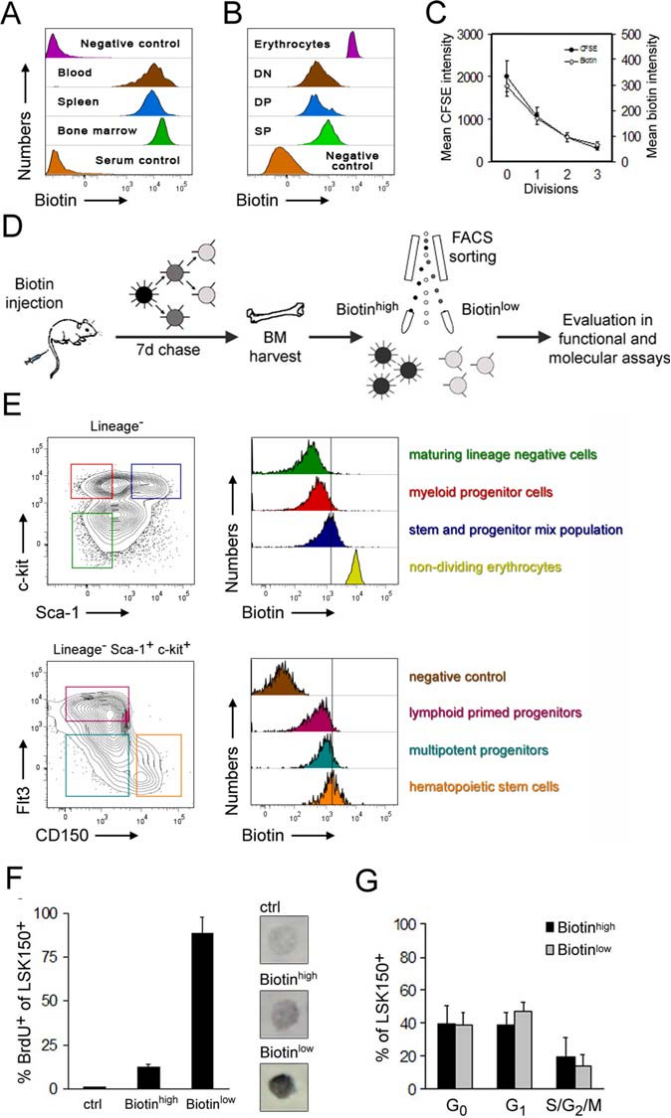
Non-invasive biotin labeling, a tool to separate cells with differential divisional kinetics. (A) Biotin label intensities on T cells from blood, spleen and bone marrow 5 minutes after intra venous injection of biotin, or on peripheral blood T cells mixed with serum (serum control) taken 15 minutes after intra venous injection of biotin (representative plots from one of 5 mice in each group). (B) Reduction of biotin label intensities on CD4/CD8 thymocyte subsets 3 days after biotin injection (DN = double negative, DP = double positive, and SP = single positive cells). Initial biotin intensities are indicated by non-dividing erythrocytes (representative plots from one of 10 mice in total). (C) Dilution kinetics of biotin in relation to the intra-vital tracking dye CFSE. Splenocytes were labeled with biotin by intra venous injection, harvested and stained with CFSE *in vitro* and subsequently transferred to sub-lethally irradiated recipients (n = 7). Following 3 days of chase, reduction in label intensities of CD8^+^CD44^+^ T cells were evaluated by FACS. (D) Experimental outline for labeling and analysis of bone marrow cells (BM) based on differential biotin label dilution. (E) Prospective isolation of hematopoietic stem and progenitor cells and evaluation of biotin dilution kinetics 7 days following intra venous biotin injection reveals augmented proliferation kinetics (biotin dilution) accompanying HSC differentiation (representative plots from one of more than 30 separate isolations). (F) BrdU label frequencies of LSKCD150^+^ cells sorted as biotin low and high onto slides for BrdU staining 7 days after biotin injection (images show differences in label intensities of representative biotin low and high BrdU positive cells). (G) Cell cycle status (determined by Ki67/DAPI stain) of LSKCD150^+^ HSCs with high and low biotin intensities 7 days after biotin injection (n = 5). All data are presented as mean (±SD).

Administration of biotin to mice resulted in complete labeling of all hematopoietic cells in peripheral blood, spleen, bone marrow (Fig. 1A), and thymus (JMN and DB, data not shown) as early as five minutes post-injection. Serum taken immediately from biotin-injected mice failed to label cells of untreated mice, demonstrating the rapid clearance of biotin from circulation (Fig. 1A).

Shortly after intravenous biotin injection, the biotin label was found to be highly uniform (Fig. 1A). By contrast, when investigated several days after labeling, individual cells exhibited significant differences in biotin label (Fig. 1B). We speculated that this difference was caused by different cell cycle rates among individual cells, such that biotin-labeled membrane proteins had been diluted to a larger extent in more proliferative cells. In an attempt to verify this hypothesis, we first labeled cells *in vivo* with biotin. Next, we harvested biotin-labeled splenocytes from these mice and co-stained them *in vitro* with the extensively utilized viable cell division tracking dye CFSE [13]. Labeled cells were next reintroduced to new mice and allowed to proliferate *in vivo* for 3 days, after which splenocytes were again harvested and evaluated for their proliferative history (Fig. 1C). In a complementary experiment CFSE labeled splenocytes were obtained from OTII mice, whose peripheral T cells are capable of antigen-dependent proliferation [14], and subjected to immunization with Ovalbumin (Fig. S1) to track proliferative history of CD4^+^ T cells. These two experiments verified that the biotin label was distributed to daughter cells with dilution kinetics comparable to that of CFSE.

Next, we wanted to assess whether biotin labeling could be used to track the *in vivo* proliferative behavior of HSCs. To this end, we first biotin-labeled cells *in vivo* and prospectively FACS purified HSCs from the bone marrow based on their unique cell surface phenotype (experimental strategy outlined in Fig. 1D). One week after biotin labeling, phenotypic HSCs were highly enriched within the fraction of bone marrow cells with highest biotin labeling (Fig. 1E). By contrast, less primitive progenitor cells had diminished biotin labels (Fig. 1E).

To validate that the reduction in biotin label was due to proliferation-induced dilution of the label in HSCs, we next investigated how well the biotin dilution correlated with DNA replication. For this purpose, mice receiving an intravenous injection of biotin were simultaneously treated with BrdU for 7 days. Candidate HSCs were sorted by flow cytometry into fractions with high and low levels of biotin labeling and then stained for BrdU content. Whereas cells with a diminished biotin label were identified as being highly proliferative (89±9% BrdU^+^), cells with high levels of biotin label had a reduced cell proliferation profile (13±2% BrdU^+^) (Fig. 1F). In similar experiments that allowed comparison of the BrdU and biotin techniques for proliferation analysis of candidate HSC we observed significant changes in proliferation rates as a consequence of high dose long term BrdU administration (Fig. S2). These observations identify an important caveat with the BrdU technique since it apparently modulates the proliferation rate of candidate HSCs *in vivo*. We next investigated the ‘snapshot’ cell cycle distributions of low- and high-biotin cells. This analysis confirmed that a significant portion of both biotin-retaining and -displacing cells were in a quiescent state at any given time, with less than 20% in cycle (Fig. 1G). This agrees with previous data demonstrating that the most primitive subset of cells in the HSC population divides with prolonged kinetics [12]. However, whereas the tracing of biotin labeling over time could easily establish differences in proliferative activity between individual candidate HSCs (Fig. 1E), these experiments highlight the need for large variations in mitotic index in order for snapshot cell cycle analyses to adequately reveal the differential proliferative behavior of rare subsets such as the candidate HSC fraction investigated here.

### Heterogeneous proliferation kinetics within a candidate HSC compartment

**Figure 2:**
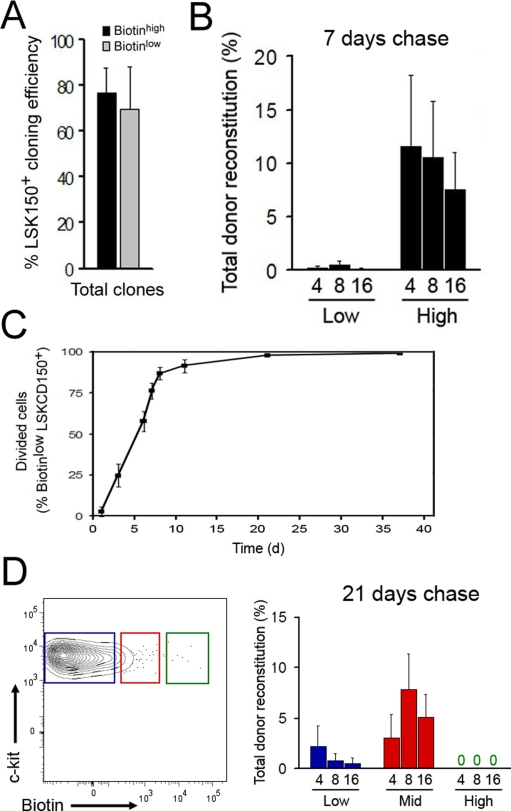
Biotin dilution kinetics reveals functional heterogeneity within HSC populations *in vivo*. (A) Single cell *in vitro* clonogenic potential (n = 4 separate experiments) of LSKCD150^+^ HSCs with differential proliferation history and (B) long-term peripheral blood cell reconstitution from competitive transplantations of 5 isolated LSKCD150^+^ HSCs following 7 days chase. Cells with fast (biotin^low^) or slow (biotin^high^) proliferation kinetics were compared (n = 7 recipients per group, data from one of three independent experiments). Numbers on x-axis represents weeks post transplantation. Similar differences were found within HSCs populations isolated using different sets of phenotypic markers including CD34 [27] and CD48 [28]. (C) Biotin dilution kinetics on LSKCD150^+^ HSC evaluated by FACS at several time points following intravenous injection of biotin. At all time points, the biotin intensity of non-dividing Ter119^+^ erythrocytes in the bone marrow was sustained at original levels (n = 5–7 mice per data point). (D) Biotin label intensities on LSKCD150^+^ HSCs after 21 days chase and total donor reconstitution frequencies from competitive transplantations of 5 biotin low (blue), intermediate (red), and high (green) HSCs (n = 4–6 recipients/group). Numbers on x-axis represents weeks post transplantation. All data are presented as mean (±SD).

Previous studies have suggested that 50–60% of all HSCs divide within 7 days, with an almost complete turnover of the HSC compartment by 30 days [3], [4]. Because HSCs analyzed in these studies were defined by phenotype alone, heterogeneity in proliferation kinetics could reflect either the presence of functionally distinct HSC subsets or differences in regulation or maintenance of quiescence in individual HSCs. We speculated that differential proliferative properties within HSC-enriched populations could be linked to changes in cellular function. To begin to address this question, we first evaluated the *in vitro* clonogenic capacity of candidate HSCs with differential proliferative histories during the course of 7 days. These experiments did not reveal any major differences associated with proliferative history (Fig. 2A). In order to assay their *in vivo* HSC properties, slow and more rapidly proliferating candidate HSCs were next competitively transplanted into lethally irradiated congenic recipients, and HSC engraftment was evaluated by their multi-lineage contribution to the peripheral blood cell pool. These experiments revealed that the slowly dividing HSC subset was superior at competitively reconstituting recipient mice in the long-term (Fig. 2B) and displayed competence at producing the full range of hematopoietic cell lineages (Fig. S3). A similar relationship was observed for their self-renewal abilities, as determined by serial transplantation of primary engrafted donor cells (Fig. S3). When performing biotin chase for longer time periods, we could establish complete turnover of all candidate HSCs within as little as three weeks (Fig. 2C), at which time no detectable HSC activity was present within candidate HSC displaying biotin intensities at original levels (Fig. 2D). These data demonstrate that isolation and functional evaluation of cells based on their past proliferation kinetics can be used to identify the most primitive subset of cells within the HSC compartment, and that changes in proliferative behavior to a large extent precede the changes in HSC phenotype that accompany loss of HSC self-renewal.

### Tracing the functional *in vivo* behavior of candidate HSCs as a consequence of stress or divisional retardation

**Figure 3:**
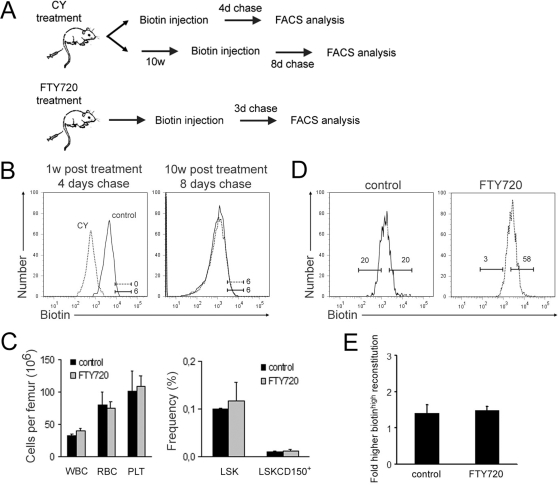
Tracing the functional *in vivo* behavior of candidate HSCs as a consequence of stress or divisional retardation. (A) Experimental outline for evaluation of Cyclophosphamide (CY) and FTY720 effects on HSC proliferation. (B) Biotin intensities following vehicle (control) or CY injections (n = 7 mice per group and time point). (C) Bone marrow cell parameters measured by a microcell counter (left), and stem cell frequencies measured by FACS (right), three days post FTY720 injections (n = 4 mice). (D) Proliferation kinetics of LSKCD150^+^ HSCs was significantly retarded by FTY720 treatment, evaluated by measuring biotin intensities three days following vehicle (control) or FTY720 injections (n = 7 mice per group, p-value = 1.18×10^−5^). (E) Fold difference in total donor reconstitution frequencies from competitive transplantations of 50 high- or low-biotin LSKCD150^+^ HSCs in one representative experiment of two (n = 10 mice per group). All data are presented as mean (±SD).

To examine HSC responses to biological stress, we next investigated their *in vivo* proliferation following the administration of the immunosuppressant cyclophosphamide (CY). Metabolites of CY form DNA crosslinks that normally induce cell death. However, toxic metabolites are not formed in HSCs and therefore the main effect of CY on HSCs is on activation to compensate for the loss of downstream progenitor cells [6]. Importantly, and in contrast to many other chemotherapeutic agents, HSC from CY treated mice retain their cell surface phenotype and functional properties [15], [16]. To study the effect of CY on HSC proliferation, we injected mice with biotin directly after CY treatment (Fig. 3A) and investigated the biotin label on HSCs after 4 days of chase. Candidate HSCs from CY-treated mice proliferated at a dramatically faster rate than candidate HSCs from vehicle-treated mice (Fig. 3B). By contrast, candidate HSCs from previously CY- and vehicle-treated mice displayed similar proliferation levels when hematopoietic parameters had approached original levels 10 weeks after CY treatment (Fig. 3B, Fig. S4). These experiments demonstrate that although cytostatic administration profoundly affects the proliferation kinetics of HSCs in the short-term, such effects are transient and therefore reversible. We furthermore could conclude that the increased rates of HSC proliferation in response to CY treatment leads to rapid and complete turnover of the whole HSC population to ensure hematopoietic recovery. Thus, we found no evidence for slow proliferating HSCs with extraordinary abilities to remain quiescent in situations of extreme stress.

It is well established that the interactions of HSCs with the bone marrow microenvironment can significantly influence their function and fate [17]. Stromal-cell-derived factor 1 (SDF-1) interacts with the CXC chemokine receptor 4 (CXCR4) on HSCs to mediate their homing to the bone marrow HSC niche. It has been proposed that one function of such niches is to mediate HSC quiescence [17]. We therefore hypothesized that enhancement of this interaction might increase HSC adherence to the bone marrow HSC microenvironment, and perhaps contribute to the maintenance of HSC quiescence. Sphingosine 1-phosphate receptors (S1PR) affect the responsiveness of HSCs and progenitor cells to chemokines, including SDF-1, resulting in enhanced homing to the bone marrow following transplantation, as well as increased adherence to stromal cells [18]. FTY720 is an immunosuppressant isolated from the ascomycete *Isaria sinclairii*. It has previously been demonstrated to act as a ligand for S1P receptors to induce homing and retention of circulating lymphocytes to lymph nodes [19]. Activation of S1PR on HSCs by repeated intra-peritoneal injection of FTY720 resulted in enhanced SDF-1 signaling in HSCs, but had no immediate effect on levels of HSC and progenitor cells in the bone marrow (Fig. 3A, C). However, comparison of the *in vivo* proliferation kinetics in FTY720-treated mice revealed that candidate HSCs had retarded proliferation kinetics when compared to vehicle-treated controls (Fig. 3D). To evaluate whether such changes could result in increased functional HSC activity, lethally irradiated mice were competitively transplanted with high- or low-biotin candidate HSC isolated from FTY720 or vehicle-treated mice. These experiments revealed that S1PR activation on candidate HSCs, which appears to induce such cells into a more quiescent state, is not sufficient to enhance HSC function (Fig. 3E). Therefore, S1PR activation appears to retard the proliferation of primitive progenitors, rather than long-term repopulating HSCs.

### Alterations in divisional kinetics mark an initial event in HSC commitment

**Figure 4:**
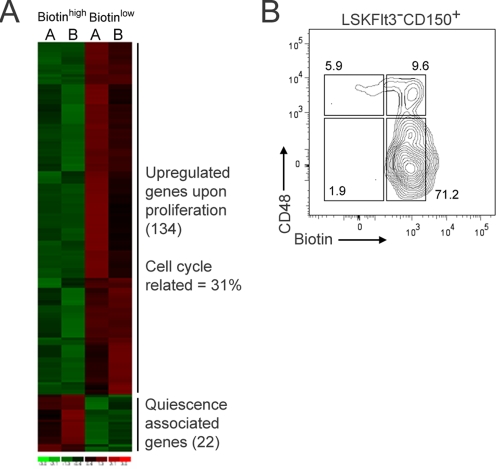
Gene expression profiling of HSCs with different proliferative kinetics, based on biotin dilution. (A) Heat map showing differential expression profiles of LSKCD150^+^ HSCs isolated as fast (biotin low) or slow (biotin high) proliferating based on differential biotin intensity 7 days after *in vivo* biotin labeling. Up-regulated genes are shown in red and down-regulated genes in green. A and B refer to biological replicates. (B) FACS analysis of CD48 protein levels (%) on LSKFlt3^−^CD150^+^ candidate HSCs in relationship to their proliferation history.

Having established a reliable assay to track *in vivo* cellular proliferation history, we next decided, as a proof-of-principle, to conduct analyses of the gene expression patterns associated with proliferation of candidate HSCs. Following biotin injection and 7 days of chase, candidate HSCs with high or low biotin retention were isolated by flow cytometry. Next, mRNA was extracted and subjected to hybridization onto Affymetrix whole genome oligonucleotide arrays. Following stringent selection criteria, these experiments revealed 156 genes (165 probe sets) to be two-fold or higher differentially expressed (Fig. 4A, Table S1), with 134 genes (142 probe sets) up-regulated upon proliferation, and 22 genes (23 probe sets) associated with quiescence/slow proliferation. Gene Ontology analysis revealed that as much as 31% of the genes up-regulated in the highly proliferating fraction were associated with cell cycle (p-value 1.86×10^−11^), and therefore recognized the cellular process underlying the differential label-retention over time using an independent and unbiased approach. With a few exceptions, most of the genes identified by this approach have not been previously investigated regarding their involvement in HSC regulation. However, when compared to previous microarray data from HSCs and their immediate progeny, we identified several common genes. Onset of CD48 expression (Fig. 4B) in HSCs has been shown to mark an early step in their commitment towards a progenitor cell stage, along with loss of stem cell properties [20]. Furthermore, and in line with previous studies, genes such as Vcam1 and Necdin, which are associated with long-term HSC identity [21], were expressed at higher levels in the more slowly dividing fraction. Based on these experiments, we conclude that *in vivo* proliferative history should be a useful marker to unravel the initial molecular differentiation events of HSCs and to identify putative novel regulators of HSC quiescence.

## Discussion

The mechanisms underlying the fates of HSCs are still largely unknown, although several descriptive models of such processes have been established. Common among these is that self-renewing and multi-potent long-term HSCs exist throughout life, maintaining quiescence relative to other hematopoietic cells [1]. HSC progeny irreversibly become more rapidly proliferating multi-potent progenitors, with an ability to contribute to multilineage hematopoiesis for no longer than 6 to 8 weeks [1]. The regulators of the cell cycle processes that underlie this scheme of differentiation remain to be determined. In doing so, there is a need for robust techniques to study cell proliferation kinetics *in vivo*.

Previous attempts to investigate HSC proliferation history have been limited to assays that involve *in vitro* manipulation of donor cell populations for efficient labeling, or that require fixation to permit experimental readout [2], [3], [5], [8], [22]. Therefore, most existing experimental data for HSC quiescence has been correlative, resting on the phenotypic identity of HSCs rather than their functional capacities. Here, we developed an *in vivo* labeling technique adapted to permit assessment of cellular proliferation history while leaving blood cell homeostasis unperturbed. We believe this is key, because although prospective identification of stem cells has advanced considerably during the last decades, with the hematopoietic system representing the forefront of such work, functionality still remains the only reliable criterion by which to define stem cell identity [1]. Furthermore, this approach offers the unique ability to probe *in vivo* the molecular features associated with more extensive proliferation. Because injection of biotin labels hematopoietic cells throughout the hematopoietic compartments, we anticipate that this technique should be useful in several other contexts where *in vivo* proliferation of hematopoietic cells is to be monitored.

**Figure 5:**
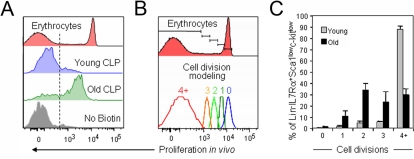
Aged common lymphoid precursors exhibit a diminished proliferative capacity *in vivo.* Young (10–12 week) and aged (100 week) mice were injected with biotin. Bone marrow was harvested 7 days later, and analyzed for cells with a common lymphoid precursor (CLP) phenotype [29] in addition to biotin label determination. (A) Biotin label profiles of erythrocytes (non-dividing control) and CLPs, as well as the biotin label profile of CLPs in the absence of biotin injection. (B) The biotin profiles of dividing and non-dividing erythrocytes were used to model individual cell divisions using the ‘Proliferation’ function in FlowJo software. (C) Young and aged CLPs were evaluated for the number of cell divisions they had undergone during 7 days using the modeling strategy described in (B). All data are presented as means (±SD); n = 5 mice per group.

There are potential caveats to this proposed technique. Compared to *in vitro* labeling approaches with compounds such as CFSE, the *in vivo* biotin technique does not yield similarly intense staining patterns. This means that the number of cell divisions that can be traced over time become more limited, although it still provide sufficient resolution for the work presented here. Furthermore, as the technique rests on the labeling of membrane proteins that segregate to daughter cells upon division, it is conceivable that some non–proliferation-associated dilution of the biotin label may occur as biotin-labeled membrane proteins with high turnover are exchanged or internalized. Similar concerns apply to established *in vitro* labeling reagents such as CFSE [23], although we believe it should not greatly affect experimental interpretation, particularly when evaluated in a comparative setting. However, this issue may be of concern when attempting to determine more accurately the number of cell divisions that individual cells have undergone during a defined time frame. Perhaps one way to obtain such information is to use non-dividing erythrocytes to define the biotin label associated with undivided cells. This population is present in all bone marrow preparations of mice receiving biotin, and can therefore be used as an internal standard to determine the biotin label dilution in individual mice. Using commercial software for flow cytometric analysis, enumeration of cell division history can next be performed to model proliferation. One example of such an analytic strategy is provided in Fig. 5. Here, we evaluated the *in vivo* proliferative history of candidate common lymphoid precursor (CLP) cells in young and aged mice during one week. *In vitro* evaluation has previously demonstrated that aged CLPs are characterized by poor mitogenic responsiveness [24]. Using the strategies outlined herein, such analyses can now be easily extended to a more physiologic *in vivo* setting.

With the described biotin labeling approach, we have found that the vast majority of candidate HSCs regularly enters the cell cycle, albeit with slower kinetics compared to other hematopoietic progenitors. The nature of this quiescence is relative rather than absolute, because we could find no evidence that HSCs lie dormant for extended time periods. Furthermore, proliferation kinetics was found to be a highly useful parameter with which to assess differences in cellular function of individual cells within a highly purified HSC subset (LSKCD150^+^; constituting only ∼0.01% of total bone marrow cells). We speculated that the *in vivo* modulation of HSC proliferation kinetics, either by CY or FTY720 administration, would affect HSC function. However, we could observe neither enhancement nor loss of HSC function despite enforced cell cycling or retention, and so we currently interpret changes in HSC proliferation in steady state as secondary effects attributable to the differentiation process, and not the initiator of commitment. Regardless, these approaches demonstrate the utility of the *in vivo* biotin labeling assay in revealing the proliferative effects of externally administered compounds that modulate *in vivo* proliferation dynamics.

Until now, it has remained uncertain as to whether a small fraction of cells within the HSC pool remain mitotically quiescent for extended periods of time. We have determined here that more than 90% of all HSCs divide within 10 days, with complete HSC turnover taking place within a three-week window. Therefore, we may conclude that label retention is not a property that uniquely defines cells with HSC properties. These observations raise questions as to whether differences in proliferative rates of HSCs and committed progenitor cells are sufficient to keep HSCs protected from exhaustion and accumulation of cellular and genetic damage throughout development.

In summary, we have developed an assay that for the first time allows for the direct demonstration that slowly dividing candidate HSCs in steady state have an improved *in vivo* multi-lineage and self-renewing capacity compared to their more rapidly proliferating counterpart. The prolonged cell divisional kinetics associated with HSCs appears to mark a defining HSC property, likely to be associated with their maintenance and self-renewal throughout life.

## Materials and Methods

### Mice and pre-treatment

Congenic C57BL/6 strains differing at the CD45 locus were used in all experiments. Experiments were approved by the ethical committee at Lund University. Mice were housed under pathogen-free conditions in individually ventilated cages. NHS-biotin (EZ-Link Sulfo-NHS-LC-LC-biotin; Pierce) was injected intravenously at 5 mg/ml in 0.9% saline (0.2 ml/mouse). FTY720 (Cayman Chemical) was reconstituted in DMSO (20 mg/ml) and aliquots were diluted for injection with 0.9% saline (0.25 mg/ml) at each injection time, and 0.1 ml/mouse was injected intravenously at three time points every second day (at the last injection time, mice were in addition injected intravenously with biotin). Cyclophosphamide (Sendoxan; Baxter) was injected intravenously (0.1 ml/mouse of 25 mg/ml in 0.9% saline) twice, with injections two days apart. Measurements of peripheral blood and bone marrow parameters were performed using a microcell counter (Sysmex).

### HSC purification

Isolation of candidate HSCs from the bone marrow of 10-14 weeks old C57BL/6 mice was performed as described previously [25], using a FACSAria (Becton Dickinson). Briefly, isolated bone marrow cells were enriched for c-kit positive cells by c-kit antibody-conjugated beads (Miltenyi) and magnetic depletion of c-kit negative cells. Enriched cells were stained with fluorochrome-conjugated antibodies specific for lineage markers (B220, CD3, Gr-1, Mac-1, Ter119), CD48, c-kit, Sca-1, CD34, Flt3, and CD150 and streptavidin for detection of *in vivo* labeled biotin. Re-analysis of sorted cells reproducibly demonstrated high purity (>98%).

### BrdU retention and cell cycle analysis

Mice were given an intraperitoneal injection of BrdU (Sigma-Aldrich) in 0.9% saline (1 mg per 6 g body weight), and drinking water containing BrdU (1 mg/ml). Seven days later, HSCs were sorted on a FACSAria using a modulated specimen holder for simultaneous 4-population sort directly onto poly-lysine coated glass slides. Following fixation and denaturation, BrdU was detected by biotinylated monoclonal antibodies (PRB-1) and ABC/DAB (Vector Laboratories). For evaluation of the effect from accumulation of BrdU on proliferation behavior of HSCs high dose treatment with BrdU was continued for 10 days (daily intraperitoneal injections of 1 mg BrdU per 6 g body weight). For cell cycle analysis, bone marrow cells were incubated with fluorophore-conjugated antibodies as described above, fixed (2% paraformaldehyde, 20 min), stained with Ki67 antibodies (in 0.1% saponin, 1h) and DAPI. All samples were analyzed on a FACSAria.

### CFSE label dilution

Comparison of CFSE and biotin label dilution kinetics was performed on T cells in spleens. Donor splenocytes (CD45.1) were labeled with biotin *in vivo* by intra venous injection and directly thereafter isolated and stained *in vitro* with CFSE (0.5 mM in PBS at room temperature for 5 minutes). After blocking with PBS supplemented with 5% FCS, 7×10^6^ splenocytes were intra venously transplanted into recipient (CD45.2) mice. After 3 days, the two types of labels were compared on donor derived CD8^+^CD44^+^ T cells in recipient spleens by FACS. Alternatively, splenocytes were obtained from CD45.1/CD45.2 OTII mice [14], CFSE stained and next transferred to CD45.2 hosts. 5 minutes post-transfer of cells, hosts were injected with biotin. The following day, mice were immunized with Ovalbumin and adjuvant, and proliferation kinetics of CD4^+^ donor derived T cells was evaluated 3 days after immunization. All samples were analyzed on a FACSAria.

### Single cell cultures

HSCs were sorted using a FACSAria (1 cell/well, 120 cells/group) in Terasaki plates (Nunc) and cultured in conditions as previously described [23]. Each well was inspected within six hours of sorting, and only wells containing one cell were included in the experiment. Wells were scored for cell growth after 10 days of culture at 37°C.

### Competitive repopulation assays

FACS-sorted HSCs from C57BL/6 mice (CD45.1) were transplanted (5–50 cells per mouse) into lethally irradiated (900 rads) congenic recipients (CD45.2) together with 200,000 unfractionated bone marrow competitor cells (CD45.2) to allow quantification of reconstitution activity and ensure survival of lethally irradiated mice. Hematopoietic donor cell reconstitution and lineage distribution was evaluated in peripheral blood at different time points post-transplantation by FACS, as previously described [25]. For secondary transplantations, lethally irradiated recipient mice were competitively transplanted with 50 LSKCD150^+^ HSCs isolated from primary recipients.

### Affymetrix gene expression analysis and quantitative RT-PCR

RNA was extracted from 2,000–3,000 HSCs sorted as high- or low-biotin with an RNeasy mRNA purification kit (Qiagen). Subsequent handling was performed by the SweGene Affymetrix unit at Lund University as previously described [26]. Probe level expression values were extracted by RMA, and analysis was performed with dChip software (http:/biosun1.harvard.edu./complab/dchip/) after filtering (0.5<SD/mean<1,000 and a baseline expression of at least 50 in 50% of samples). Genes with two-fold or higher expression differences were identified (at 90% or higher confidence interval).

### Statistics

All data are reported as mean±SD. Statistical comparisons were made using Student's t-test for unpaired samples and differences with P<0.05 were regarded as significant.

## Supporting Information

Figure S1 CFSE and biotin label intensities are similarly reduced following cell division. (A) 5×10^6^ splenocytes from CD45.1/CD45.2 OTII donor mice were stained with CFSE *in vitro* and thereafter transplanted to CD45.2 recipients. Donor and recipient cells were labeled with biotin *in vivo* 5 minutes post cell transfer, and label dilutions were analyzed on donor CD4^+^ T cells 3 days following immunization with Ovalbumin. (B–C) Representative plots of CFSE and biotin label dilution on T cells in recipient spleens (n = 3).
(0.85 MB TIF)

Figure S2 Effect on proliferation of candidate HSC following BrdU treatment *in vivo*. (A) Mice received intra peritoneal injections with BrdU or PBS for 10 days prior to analysis. On day 3 after start of BrdU injections, biotin was injected intravenously. The proliferation effect from BrdU accumulation in LSKCD150^+^CD34^−^ HSCs was determined by FACS analysis of biotin intensities. (B) Using the 'Proliferation' function in FlowJo software, the biotin profile of erythrocytes (non-dividing cells) was used to model the number of cell divisions that individual LSKCD150^+^CD34^−^ HSCs had undergone in either PBS injected or BrdU injected mice. Graphs depicts % of HSCs (y-axis) having undergone the defined number of cell divisions (y-axis). Black bars show HSCs proliferation in PBS treated mice and grey bars show HSC proliferation in BrdU treated mice. All data are presented as means (±SD); n = 5 mice per group. Asterisks indicate p-values lower than 0.05.
(0.94 MB TIF)

Figure S3 Biotin dilution kinetics reveals heterogeneity of self-renewing properties and multi-potency in HSC populations *in vivo*. (A) Sort gates for isolation of LSKCD150^+^ HSC from mice 7 days after intra venous biotin injection (left panel). Re-analysis of fast (biotin low) and slow (biotin low) proliferating HSCs based on differential distribution of biotin intensities (right panel). Brackets in left panel represents intensity of non-injected negative control LSKCD150^+^ HSCs (left brackets) and positive control LSKCD150^+^ HSCs isolated five minutes after biotin injection (right brackets). (B) Total donor peripheral blood reconstitution frequencies in primary recipients after competitive transplantations of 50 biotin high or low LSKCD150^+^ HSCs isolated after 7 days chase. Secondary recipients were competitively transplanted with 50 LSKCD150^+^ HSCs isolated from primary recipients. Numbers on x-axis represents weeks post transplantation. Data are from one representative experiment of five (45 mice in each group in total). (C) Distribution of total donor reconstituting cells from competitive transplantations of 50 biotin high or low LSKCD150^+^ cells into B cells (B220^+^), T cells (CD4^+^ and/or CD8^+^) and myeloid cells (CD11b^+^). Numbers on x-axis represents weeks post transplantation. Data are from the same experiment as in (B) and are presented as mean (±SD).
(2.67 MB TIF)

Figure S4 Hematopoietic parameters following Cyclophosphamide (CY) treatment (A) Total bone marrow cells measured by a microcell counter (left), and candidate HSC frequencies measured by FACS (right), one week after CY injections. (B) Body weight and peripheral blood cell levels over time in vehicle (control) and CY-injected mice. Numbers on x-axis represents weeks post CY injections. Asterisks represent significant differences compared to respective control group (n = 7 mice per group and time point). All data are presented as mean (±SD).
(0.52 MB TIF)

Table S1 Genes and probe sets differentially expressed in LSKCD150^+^ HSC isolated as fast proliferating (biotin low) or slow proliferating (biotin high).
(0.02 MB PDF)
