# Supplementary figures and images for: A Novel Assay to Trace Proliferation History In Vivo Reveals that Enhanced Divisional Kinetics Accompany Loss of Hematopoietic Stem Cell Self-Renewal
Source: PLoS One. 2008 Nov 12;3(11):e3710. doi: 10.1371/journal.pone.0003710 (PMC2580029; doi:10.1371/journal.pone.0003710)

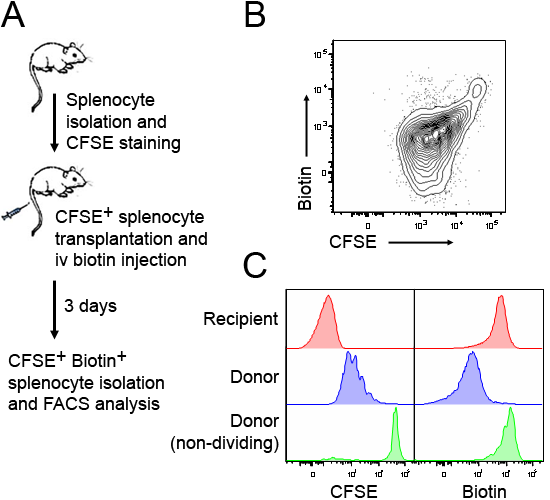

Supplement: Figure S1 — CFSE and biotin label intensities are similarly reduced following cell division. (A) 5×10^6^ splenocytes from CD45.1/CD45.2 OTII donor mice were stained with CFSE in vitro and thereafter transplanted to CD45.2 recipients. Donor and recipient cells were labeled with biotin in vivo 5 minutes post cell transfer, and label dilutions were analyzed on donor CD4+ T cells 3 days following immunization with Ovalbumin. (B–C) Representative plots of CFSE and biotin label dilution on T cells in recipient spleens (n = 3). (0.85 MB TIF) [file pone.0003710.s001.tif]

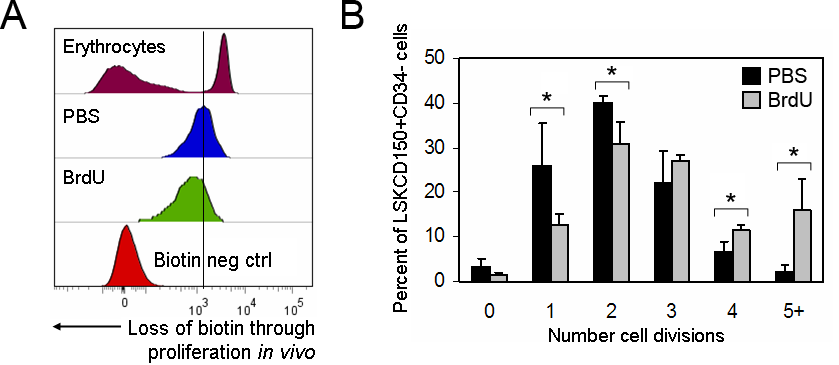

Supplement: Figure S2 — Effect on proliferation of candidate HSC following BrdU treatment in vivo. (A) Mice received intra peritoneal injections with BrdU or PBS for 10 days prior to analysis. On day 3 after start of BrdU injections, biotin was injected intravenously. The proliferation effect from BrdU accumulation in LSKCD150+CD34− HSCs was determined by FACS analysis of biotin intensities. (B) Using the 'Proliferation' function in FlowJo software, the biotin profile of erythrocytes (non-dividing cells) was used to model the number of cell divisions that individual LSKCD150+CD34− HSCs had undergone in either PBS injected or BrdU injected mice. Graphs depicts % of HSCs (y-axis) having undergone the defined number of cell divisions (y-axis). Black bars show HSCs proliferation in PBS treated mice and grey bars show HSC proliferation in BrdU treated mice. All data are presented as means (±SD); n = 5 mice per group. Asterisks indicate p-values lower than 0.05. (0.94 MB TIF) [file pone.0003710.s002.tif]

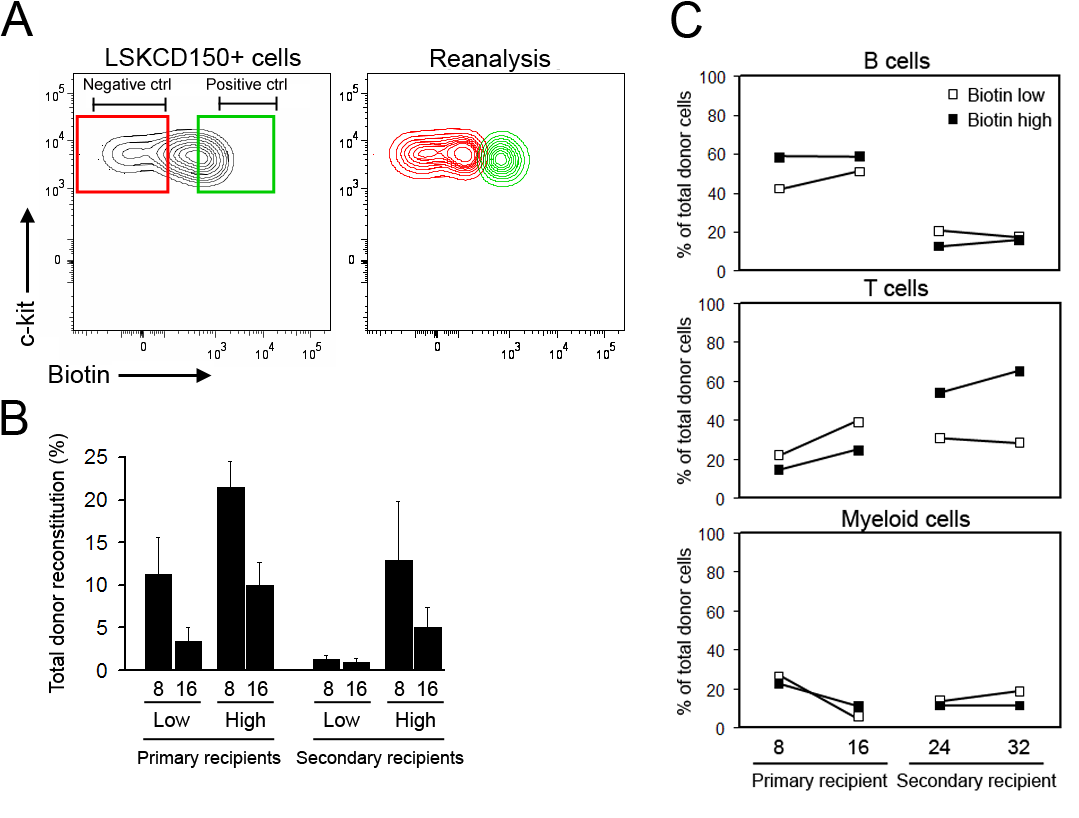

Supplement: Figure S3 — Biotin dilution kinetics reveals heterogeneity of self-renewing properties and multi-potency in HSC populations in vivo. (A) Sort gates for isolation of LSKCD150+ HSC from mice 7 days after intra venous biotin injection (left panel). Re-analysis of fast (biotin low) and slow (biotin low) proliferating HSCs based on differential distribution of biotin intensities (right panel). Brackets in left panel represents intensity of non-injected negative control LSKCD150+ HSCs (left brackets) and positive control LSKCD150+ HSCs isolated five minutes after biotin injection (right brackets). (B) Total donor peripheral blood reconstitution frequencies in primary recipients after competitive transplantations of 50 biotin high or low LSKCD150+ HSCs isolated after 7 days chase. Secondary recipients were competitively transplanted with 50 LSKCD150+ HSCs isolated from primary recipients. Numbers on x-axis represents weeks post transplantation. Data are from one representative experiment of five (45 mice in each group in total). (C) Distribution of total donor reconstituting cells from competitive transplantations of 50 biotin high or low LSKCD150+ cells into B cells (B220+), T cells (CD4+ and/or CD8+) and myeloid cells (CD11b+). Numbers on x-axis represents weeks post transplantation. Data are from the same experiment as in (B) and are presented as mean (±SD). (2.67 MB TIF) [file pone.0003710.s003.tif]

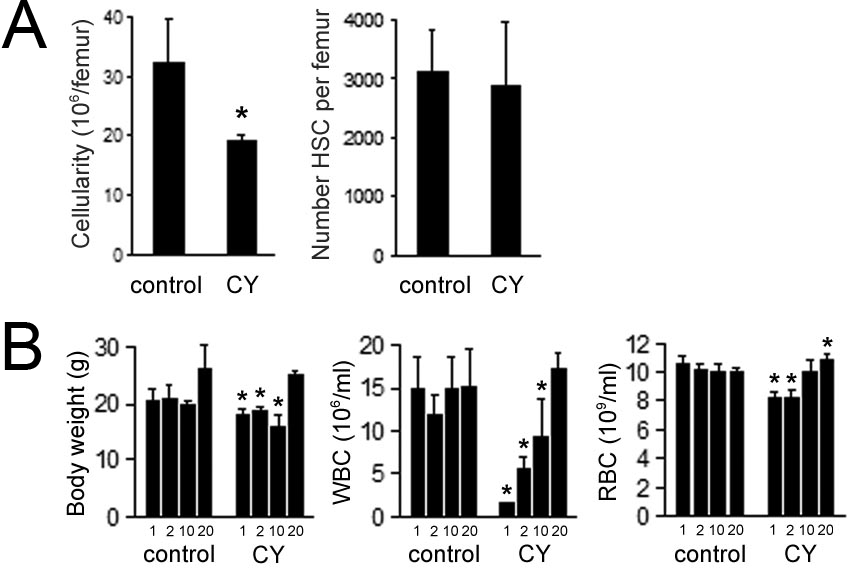

Supplement: Figure S4 — Hematopoietic parameters following Cyclophosphamide (CY) treatment (A) Total bone marrow cells measured by a microcell counter (left), and candidate HSC frequencies measured by FACS (right), one week after CY injections. (B) Body weight and peripheral blood cell levels over time in vehicle (control) and CY-injected mice. Numbers on x-axis represents weeks post CY injections. Asterisks represent significant differences compared to respective control group (n = 7 mice per group and time point). All data are presented as mean (±SD). (0.52 MB TIF) [file pone.0003710.s004.tif]
