# Supplementary material for: A Novel Assay to Trace Proliferation History In Vivo Reveals that Enhanced Divisional Kinetics Accompany Loss of Hematopoietic Stem Cell Self-Renewal
Source: PLoS One. 2008 Nov 12;3(11):e3710. doi: 10.1371/journal.pone.0003710 (PMC2580029; doi:10.1371/journal.pone.0003710)
Supplement: Table S1 — Genes and probe sets differentially expressed in LSKCD150+ HSC isolated as fast proliferating (biotin low) or slow proliferating (biotin high). (0.02 MB PDF) [file pone.0003710.s005.pdf]

**Supplementary Table 1.** Genes and probe sets differentially exressed in LSKCD150$^+$ HSC isolated as fast proliferating (biotin low) or slow proliferating (biotin high)

## Upregulated genes upon proliferation

Tmem168: transmembrane protein 168
Rest: RE1-silencing transcription factor
Nt5dc2: 5'-nucleotidase domain containing 2
Ylpm1: YLP motif containing 1
Uchl3 /// Uchl4: ubiquitin carboxyl-terminal esterase L3 /// L4
EG665081: predicted gene, EG665081
Sell: selectin, lymphocyte
Mcm3: minichromosome maintenance deficient 3
Polr3c: polymerase (RNA) III (DNA directed) polypeptide C
Cdca5: cell division cycle associated 5
Cdca8: cell division cycle associated 8
Cct3: chaperonin subunit 3 (gamma)
Hells: helicase, lymphoid specific
BC055324: cDNA sequence BC055324
2810457I06Rik: RIKEN cDNA 2810457I06 gene
Chek1: checkpoint kinase 1 homolog (S. pombe)
Plek: pleckstrin
Nek2: NIMA (never in mitosis gene a) - expressed kinase 2
Polb: polymerase (DNA directed), beta
Ube2c: ubiquitin-conjugating enzyme E2C
Prc1: protein regulator of cytokinesis 1
C79407: expressed sequence C79407
Thoc3: THO complex 3
B230333C21Rik: RIKEN cDNA B230333C21 gene
Slc14a1: solute carrier family 14 (urea transporter), member 1
Add3: adducin 3 (gamma)
Cdca5: cell division cycle associated 5
Ncor1: nuclear receptor co-repressor 1
Pnn: pinin
Prpf4b: PRP4 pre-mRNA processing factor 4 homolog B
Coq7: demethyl-Q 7
BC005512 /// hypothetical protein LOC670270
Gnptab: N-acetylglucosamine-1-phosphate transferase
2600005O03Rik: RIKEN cDNA 2600005O03 gene
Tgfb1: transforming growth factor, beta 1
Plac8: placenta-specific 8
Ndc80: NDC80 homolog, kinetochore complex component
Ccdc34: coiled-coil domain containing 34
Mcm5: minichromosome maintenance deficient 5
Mphosph1: M-phase phosphoprotein 1
Cd48: CD48 antigen
Nuf2: NUF2, NDC80 kinetochore complex component
Mm.212854.1
Mast4: microtubule associated serine/threonine kinase 4
Nkg7: natural killer cell group 7 sequence
D2Ertd750e: DNA segment, Chr 2, ERATO Doi 750
Plek: pleckstrin
Lgals9: lectin, galactose binding, soluble 9
Itgb1: integrin beta 1 (fibronectin receptor beta)
Tuba8: tubulin, alpha 8
Bex6: brain expressed gene 6
Sox6: SRY-box containing gene 6
Kif2c: kinesin family member 2C
Gp5: glycoprotein 5 (platelet)
Mm.22337.1
Runx3: runt related transcription factor 3
5730590G19Rik: RIKEN cDNA 5730590G19 gene
Top2a: topoisomerase (DNA) II alpha
Chek1: checkpoint kinase 1 homolog (S. pombe)
Rbbp4: retinoblastoma binding protein 4
5730593F17Rik: RIKEN cDNA 5730593F17 gene
Fzr1: fizzy/cell division cycle 20 related 1
Cdc6: cell division cycle 6 homolog
Fen1: flap structure specific endonuclease 1
Prc1: protein regulator of cytokinesis 1
Ercc6l: excision repair cross-complementing 6
Sell: selectin, lymphocyte
Rrp12: ribosomal RNA processing 12 homolog
Optn: optineurin
E030041M21Rik: RIKEN cDNA E030041M21 gene
Utx: ubiquitously transcribed tetratricopeptide repeat gene
Atp10a: ATPase, class V, type 10A
Cpa3: carboxypeptidase A3, mast cell
Lin7c: lin-7 homolog C (C. elegans)
6530415H11Rik: RIKEN cDNA 6530415H11 gene
Snx6: sorting nexin 6
Ccdc99: coiled-coil domain containing 99
4933435A13Rik: RIKEN cDNA 4933435A13 gene
Lima1: LIM domain and actin binding 1
Top2a: topoisomerase (DNA) II alpha
Diap3: diaphanous homolog 3
Cenpf: centromere protein F
Mars: methionine-tRNA synthetase
Uhrf1: ubiquitin-like, containing PHD and RING finger domains, 1
Baz1a: bromodomain adjacent to zinc finger domain 1A
Blm: Bloom syndrome homolog (human)
1110018G07Rik: RIKEN cDNA 1110018G07 gene
Coro7: coronin 7
E2f7: E2F transcription factor 7
Snx30: sorting nexin family member 30
Me2: malic enzyme 2, NAD(+)-dependent, mitochondrial
Kntc1: kinetochore associated 1
Cdca8: cell division cycle associated 8
Cdca2: cell division cycle associated 2
Incenp: inner centromere protein
Gtf2e1: general transcription factor II E, polypeptide 1
Nup50: nucleoporin 50
5830417I10Rik: RIKEN cDNA 5830417I10 gene
Ncapd3: non-SMC condensin II complex, subunit D3
BC032204: cDNA sequence BC032204
Emilin2: elastin microfibril interfacer 2
Gpr97: G protein-coupled receptor 97
Itga2b: integrin alpha 2b
Rbpj: recomb. signal binding protein for imm. kappa J region
Skp2: S-phase kinase-associated protein 2 (p45)
Pphln1: periphilin 1
Rdh11: retinol dehydrogenase 11
Taf5: TAF5 RNA polymerase II, TATA box binding protein
Mm.211236.1
Dph4: DPH4 homolog (JJJ3, S. cerevisiae)
Gins4: GINS complex subunit 4 (Sld5 homolog)
Cul5: cullin 5
Trpc2: transient receptor potential cation channel, subfamily C
Transcribed locus
Mm.168959.1
Zdhhc13: Zinc finger, DHHC domain containing 13
Ccl27: chemokine (C-C motif) ligand 27
3110048E14Rik: RIKEN cDNA 3110048E14 gene
Zwilch: Zwilch, kinetochore associated, homolog
Uck2: Uridine-cytidine kinase 2
Otud7b: OTU domain containing 7B
4633401B06Rik: RIKEN cDNA 4633401B06 gene
Zfp472: zinc finger protein 472
Clasp1: CLIP associating protein 1
Mns1: meiosis-specific nuclear structural protein 1
Aff3: AF4/FMR2 family, member 3
Hspa4l: heat shock protein 4 like
Pus7: pseudouridylate synthase 7 homolog
Lonp1: lon peptidase 1, mitochondrial
Nipbl: Nipped-B homolog (Drosophila)
5430440L12Rik: RIKEN cDNA 5430440L12 gene
Ap2b1: adaptor-related protein complex 2, beta 1 subunit
Dnajc3a: DnaJ (Hsp40) homolog, subfamily C, member 3A
Mm.37417.1
Rgs12: regulator of G-protein signaling 12
Klhl28: kelch-like 28 (Drosophila)
Ncaph: non-SMC condensin I complex, subunit H
Gm1964: gene model 1964, (NCBI)
Mbnl3: muscleblind-like 3 (Drosophila)
Lrriq2: leucine-rich repeats and IQ motif containing 2
Aspm: asp (abnormal spindle)-like, microcephaly associated
Clspn: claspin homolog (Xenopus laevis)

## Quiescence associated genes

Gtl2: GTL2, imprinted maternally expressed untranslated mRNA
LOC665193: hypothetical protein LOC665193
ENSMUSG00000073981: predicted gene
Procr: protein C receptor, endothelial
Fscn1: fascin homolog 1, actin bundling protein (S. purpuratus)
Mrp63: mitochondrial ribosomal protein 63
Slc40a1: solute carrier family 40 (iron-regulated transporter) 1
Gprin2: G protein regulated inducer of neurite outgrowth 2
Maf: avian musculoaponeurotic fibrosarcoma (v-maf)
Tox: Thymocyte selection-associated HMG box gene
H2-Aa: Histocompatibility 2, class II antigen A, alpha
Mpa2l: macrophage activation 2 like
Alg13: asparagine-linked glycosylation 13 homolog
Ctss: cathepsin S
Ndn: necdin
Evc2: Ellis van Creveld syndrome 2 homolog (human)
Raph1: Ras association and pleckstrin homology domains 1
H2-Aa: histocompatibility 2, class II antigen A, alpha
Elac1: elaC homolog 1 (E. coli)
Gmpr2: guanosine monophosphate reductase 2
Calm3: Calmodulin 3
Marveld1: MARVEL (membrane-associating) domain 1
Vcam1: vascular cell adhesion molecule 1
